# Ultrasonographic Evaluation of Intestinal Fibrosis and Inflammation in Crohn’s Disease. The State of the Art

**DOI:** 10.3389/fphar.2021.679924

**Published:** 2021-04-26

**Authors:** Francesca Ferretti, Rosanna Cannatelli, Sandro Ardizzone, Jeanette A. Maier, Giovanni Maconi

**Affiliations:** ^1^ Gastroenterology Unit—ASST Fatebenefratelli-Sacco, Department of Biomedical and Clinical Sciences (DIBIC) L. Sacco, Università degli Studi di Milano, Milan, Italy; ^2^ General Pathology—Department of Biomedical and Clinical Sciences (DIBIC) L. Sacco, Università degli Studi di Milano, Milan, Italy

**Keywords:** Crohn’s disease, fibrosis, inflammation, strictures, ultrasound, sonoelastography

## Abstract

The evaluation of the degree of inflammation and fibrosis, intrinsic elements in intestinal wall damage of Crohn’s disease, is essential to individuate the extent of the lesions and the presence of strictures. This information will contribute to the choice of the appropriate therapeutic approach, the prediction of the response to therapy and the course of the disease. The accurate evaluation of the extent and severity of inflammation and/or fibrosis in Crohn’s disease currently requires histopathological analysis of the intestinal wall. However, in clinical practice and research, transmural assessment of the intestinal wall with cross sectional imaging is increasingly used for this purpose. The B-mode ultrasonograhic characteristics of the intestinal wall, the assessment of its vascularization by color Doppler and I.V. contrast agents, and the evaluation of the mechanical and elastic properties by sonoelastography, may provide useful and accurate information on the severity and extent of inflammation and intestinal fibrosis in Crohn’s disease. The purpose of this review is to provide an update on current sonographic methods to discriminate inflammation and fibrosis in Crohn’s disease.

## Introduction

Crohn’s disease (CD) is characterized by chronic inflammation and progressive fibrosis. In general, the inflammatory pattern is the most frequent at the onset of the disease, while a stricturing or penetrating evolution can be observed later on (Cosnes et al., 2002).

The complicated course of disease occurs in up to 50% of patients within 20 years of diagnosis, requiring a surgical intervention in half of these cases within 10 years (Peyrin-Biroulet et al., 2010).

The evaluation and characterization of inflammation is pivotal in the detection of the disease, in the characterization of newly diagnosed of CD, and in its monitoring, along with the evaluation of complications (Maaser et al., 2019). The definition of disease activity and onset of fibrosis will shape therapeutic decisions, with prognostic impact on the response to therapy and the risk of recurrence.

Histopathology is still the gold standard for the identification of inflammation and fibrosis. However, despite several scoring systems have been proposed, in particular for strictures, a wide heterogeneity in scoring methods exists and none can be considered as a suitable benchmark (Bettenworth et al., 2019; De Voogd et al., 2020; Gordon et al., 2020). In clinical practice and research, cross-sectional imaging techniques have been proposed as reliable non-invasive alternative approaches for transmural assessment of the intestinal wall to detect inflammatory or fibrotic patterns.

Among different imaging techniques, intestinal ultrasound (IUS) is a radiation-free and cost-effective technique which is increasingly adopted both for the diagnosis and the monitoring of CD. The introduction of small intestine contrast ultrasonography (SICUS) and the contrast-enhanced ultrasound (CEUS) as well as elastography expanded the potential applications of this technique.

However, reliability of IUS in detecting inflammation and fibrosis is still under investigation, as well as its applicability and usefulness in clinical practice to drive the most appropriate treatment. Most of these gaps rely on the absence of reproducible gold standards for evaluating inflammation and fibrosis to be used for complicated and not complicated patients. In fact, if the assessment of pathological features is still awaiting valid and reproducible parameters, the discrimination of fibrosis and inflammation in non-operated patients is mainly indirect and relies essentially on clinical parameters such as biochemical tests and the response to steroid or biologic treatment.

In this review we evaluated the reliability of IUS in assessing inflammation and fibrosis, mainly focusing on the available studies that have used pathological features or the response to treatment as direct and indirect reference parameters.

## Intestinal Ultrasound and Inflammation

### Applications

The identification and characterization of inflammatory features in the small bowel and/or colon are pivotal both for the diagnosis and the monitoring of IBD. The gold standard in the assessment of bowel inflammatory involvement is histology, but non-invasive approaches are demanded to limit the risks and the costs of repeated endoscopic procedures, considering also the limitation of histology in case of inadequate or superficial biopsy sampling. Thus, different non-invasive approaches have been introduced with different degrees of reliability.

Biomarkers such as C reactive protein (CRP) and fecal calprotectin (FC) are useful for monitoring IBD (Vos et al., 2013) although with some risk of false positive results (Maaser et al., 2019). Thus, the available imaging techniques including IUS, computed tomography (CT) and magnetic resonance imaging (MRI) have been increasingly applied to IBD. In a systematic review, Panes et al. compared the diagnostic accuracy of different cross sectional imaging techniques showing a high accuracy both for the CD diagnosis and CD monitoring, with good reliability for assessing disease severity and complications (Panés et al., 2011). If multiple options are available with comparable local expertize, IUS can be considered the first choice due to its non-invasiveness, low costs and repeatability (Calabrese et al., 2016).

#### Identification and Characterization of Disease Activity in Suspect Crohn’s Disease

In case of suspect CD, IUS has shown a 80% sensitivity and 97% specificity for the CD diagnosis when compared with reference standards including clinical evaluation, endoscopy, histology and/or radiology (Calabrese et al., 2016).

The accuracy of US depends on the location and severity of CD (Hollerbach et al., 1998; Astegiano et al., 2001; Parente et al., 2003). In comparison with endoscopy, cross-sectional techniques are less accurate to detect mild inflammatory luminal lesions (Panés et al., 2011).

In suspected involvement of small bowel (SB), IUS and SB capsule endoscopy (SBCE) yield similar findings. Indeed, IUS shows 72% of sensitivity and 84% of specificity (Carter et al., 2018), which can be slightly increased using oral contrast agents (e.g., SICUS) (Kopylov et al., 2017), in particular for the assessment of proximal SB lesions (Petruzziello et al., 2010).

Finally, a comparison of the diagnostic accuracy between MRI and IUS in detecting active CD—defined as the presence of ulcerations at endoscopy, CRP >8 mg/L, Calprotectin >250 μg/g, histological evidence of activity—demonstrated a lower sensitivity of IUS vs. MRI in active SB disease (90 vs. 96%, *p* 0.01) and a comparable sensitivity in case of active colonic disease (66 vs 63%, *p* 0.7) (Taylor et al., 2018).

#### Monitoring of Disease Activity in Known Crohn’s Disease

The role of IUS in the evaluation of disease activity is still controversial (Calabrese et al., 2016). In a systematic review Rimola et al. investigated the accuracy of different cross-sectional imaging techniques in assessing disease activity, and found that four US-based parameters (wall thickening, Doppler signal, loss of stratification and reduced peristalsis/compressibility) showed good correlation with endoscopic reference standard (Rimola et al., 2012). MRI showed a good or very good agreement with endoscopic or histologic reference standard when limited small bowel tracts were analyzed, while the results about colonic CD assessment were more conflicting, mainly because of the inconstant use of colonic intraluminal contrast (Rimola et al., 2012). All the MRI-based indexes included wall thickness (in mm) and the degree of wall enhancement after intravenous contrast injection. To date, magnetic resonance index of activity (MaRIA) (Rimola et al., 2011) and Clermont score (Buisson et al., 2015; Buisson et al., 2017) are the two main indexes in the assessment and grading of CD severity and mucosal lesions, evaluating the presence of ulcerations, edema, bowel thickness and relative contrast enhancement (MaRIA) or apparent diffusion coefficient (Clermont). Both have been validated compared with endoscopy, both in ileal and colonic CD. Even if data are conflicting about the concordance and degree of accuracy of these scores, a recent study demonstrated a high and equal efficacy in the detection of mucosal healing (Buisson et al., 2017), showing a substantial accuracy in the detection of endoscopic ulcerations with high specificity (81–82%) and high negative predictive value (82% NPV), while sensitivity and positive predictive values were moderate. Likewise, in a recent study by Yuksel et al., IUS showed a comparable accuracy to MRE in detecting endoscopic activity (Yuksel et al., 2019) where in endoscopically active disease, the most frequent IUS signs were increased BWT (>3 mm) and fibrofatty proliferation (not defined in the study). These findings are in keeping with another study by Livne et al., where IUS parameters, and in particular terminal ileum thickness and mesenteric fat hypertrophy, showed a significant correlation with the MaRIA score (Livne et al., 2020).

However, the main limitation and difficulties in obtaining a conclusion regarding IUS assessment of disease activity are the choice of the IUS parameters and/or their combination (e.g., IUS scores) and the choice of the reference standard adopted to assess activity, whether clinical (i.e. elevated CDAI), biochemical (CRP, fecal calprotectin), radiological and/or histological.

### Techniques and Parameters

#### B-Mode Ultrasound and Color Doppler Imaging

The data in the literature agree on the fact that main parameters suggesting inflammation and disease activity in CD are bowel wall thickening (BWT), color Doppler imaging (CDI), and bowel wall stratification (BWS), along with mesenteric fat hypertrophy and regional lymphadenopathy (Livne et al., 2020). However, these evidences come mostly from the changes of these parameters in response to the therapy.

The definition of the main parameters linked to inflammation has been indirectly obtained by assessing the behavior of the same bowel wall parameters, which result mostly affected, after biologic or corticosteroid treatment and by correlating each feature of bowel wall with the clinical (i.e. elevated CDAI, HBI) and biochemical (CRP, calprotectin) parameters (Goodsall et al., 2020). A large German multi-center study showed the improvement of different IUS parameters (BWT, parietal vascularization assessed by color-Doppler grade, lack of bowel stratification and creeping fat and mesenteric lymph nodes) after pharmacological treatment (Kucharzik et al., 2017).

The fact that BWT is one of the main parameters correlated with inflammation has also been suggested by studies showing that transmural healing, namely the normalization of the BWT (e.g., <3 mm), is strongly correlated with mucosa healing, and recent studies have shown a normalization of the thickened wall in up to 25% of patients treated with anti-TNFα drugs (Castiglione et al., 2013). A good concordance of BWT with other diagnostic studies was demonstrated both with ileocolonoscopy and MRI (Castiglione et al., 2013; Castiglione et al., 2017), thus showing the potential role of IUS in monitoring CD patients, even in pediatric age (Civitelli et al., 2016).

The available studies correlating US and histologic features define a hypoechoic echo pattern, namely the absence or disruption of the regular bowel wall stratification, in case of prevalent inflammatory stenosis (Maconi et al., 2003) and color Doppler US abnormalities of bowel wall (Sasaki et al., 2014a; Sasaki et al., 2014b).

A recent study of Bhatnagar et al. evaluates different mural and extramural US features as potential imaging predictors of histologic inflammation by comparing IUS findings with resection specimens and confirms a significant association of bowel wall and mucosal layer thickness with acute inflammation, while mesenteric fat echogenicity correlates with chronic inflammation (Bhatnagar et al., 2021). A retrospective study measured vascularity by means of color Doppler, using the Limberg score to grade the presence of blood flow within and around the bowel wall as an index of activity and inflammation, and compared the findings with the histologic results obtained on ileum biopsies of 32 CD patients. However, the association between the Limberg score and histologic grades of disease activity was poor (κ = 0.4375) (Drews et al., 2009).

More recently, an ultrasensitive ultrasound microvessel imaging (UMI) technique, able to provide a significant higher sensitivity in depicting smaller vessels compared with conventional Doppler, has been developed and compared to CT/MRI, in a pilot study of Gong et al.(Gong et al., 2020). Altered vascularization was demonstrated in case of severe inflammation, while no significant difference was found between quiescent and mild CD.

Taken into account the data of the literature, an expert consensus on inflammatory activity parameters, combined with a blinded agreement study, showed that BWT is the most important predictor of disease activity for ultrasound with a very high inter-rater agreement and correlation with overall assessment of disease activity. CDI, inflammatory mesenteric fat and BWS, which are important parameters, show moderate or nearly moderate agreement (Novak et al., 2020).

Different US activity scoring systems have been developed so far. A recent systematic review identified different available scoring systems created on the basis of endoscopic, radiologic or histologic reference standards. However, a suboptimal methodology was observed in these studies, suggesting the need for further evaluations to identify a more reliable and standardized index (Bots et al., 2018).

#### Small Intestine Contrast Ultrasonography (SICUS)

SICUS is a sonographic technique where the evaluation of the small bowel is performed before and after the ingestion of an oral contrast solution (approximately 500 ml of polyethylene glycol solution, PEG). It has been used for the detection and characterization of SB lesions and complications (Pallotta et al., 2012), in particular with a better accuracy than transabdominal US and comparable to radiologic examination (Calabrese et al., 2005; Pallotta et al., 2005). However, an adequate validation for activity monitoring compared with endoscopic score is not available. The actual scores (i.e., SLIC: sonographic lesion index for CD) have been compared to clinical activity (CDAI and CRP levels) and used in the evaluation of the response after induction or maintenance with anti-TNFα therapy (Calabrese et al., 2012; Zorzi et al., 2014). According to this small prospective study, a significant improvement of SLIC was observed after the induction period. Moreover, SICUS-responders to anti-TNFα agents have shown a better long-term outcome in terms of need for surgery, hospitalization rate and use for steroids (Zorzi et al., 2020).

#### Contrast-Enhanced Ultrasound

Unlike Doppler ultrasound, CEUS is able to detect slow-moving blood flow in small vessels, also in deep-lying bowel wall segments, and can provide qualitative and quantitative evaluations of vascularity and perfusion of the bowel walls. CEUS is performed after the injection of microbubbles (average diameter of 2.5 µm) in the blood stream. The US signals reflected by microbubbles can be easily separated from tissue signals by the softwares of the sonographic machines, and be used to discriminate vascular from avascuar tissues (e.g., to separate phlegmon from abscesses) and to quantify vascularity within the specific tissues. The latter can be assessed in a dynamic qualitative or semiqualitiative way, according to pattern of enhacement (e.g. absence/presence of enhancement of different wall layers), contrast quantification of peak intensity, or using parameters of dynamic CEUS intensity changes over time (e.g. time-intensity curves). In this setting, the application of CEUS in the evaluation of inflammation and disease activity is raising great interest. Migaleddu et al. reported a 93.5% sensitivity and 93.7% specificity of CEUS enhancement patterns in detecting inflammatory activity with a strong correlation with CDAI in 47 CD patients. CEUS accuracy was higher than US or Color Doppler US (Migaleddu et al., 2009).

Besides the pattern of enhancement of the bowel wall, defined as the description of the arrangement of the enhanced layer, Serra et al. quantified the perfusion by using a quantitative ratio between the major thickness of the enhanced layer (E) and the thickness of the entire wall section (W) (Serra et al., 2007). According to this study, an altered pattern of enhancement and a E/W ratio ranging between 0.43 and 0.47 identified active patients with a sensitivity 81% and specificity of 55–63% (Serra et al., 2007).

The perfusion analysis with CEUS has demonstrated a prognostic value in pharmacologically treated patients. Quaia et al. identified different CEUS parameters, including the overall vascularization assessed by the area under the time-intensity curve, to discriminate responders from non-responders in newly diagnosed and treated CD patients after 3 months of therapy (Quaia et al., 2013). Moreover, Saevik et al. showed that, in case of acute exacerbation requiring anti-TNFα drugs or steroids, an high relative perfusion and thickened proper muscle layer at 1 month was predictive of treatment failure (Saevik et al., 2014). Ripolles et al.(Ripollés et al., 2009) demonstrated that an increase of 46% as the threshold brightness value has a 96% sensitivity and 73% specificity in predicting a moderate-to-severe inflammation by endoscopy in 53 CD patients. Moreover, the association was confirmed even when compared with histopathology. Indeed, Ripolles et al. showed a significant association between increased contrast enhancement (CE) and histologic inflammatory score in 28 analyzed segments (Ripollés et al., 2013).

However, comparisons among CEUS and histopathology findings are limited. In a small prospective study, Wilkens et al. compared US, CEUS and MRE parameters with histopathology of 25 patients with small bowel CD undergoing elective surgery, confirming the role of bowel wall thickness as a marker of inflammation, but reporting the failure of CEUS in discriminating between inflammatory and fibrotic lesions (Wilkens et al., 2018).

CEUS has been applied also for the post-surgery recurrence. Paredes et al. developed and applied an activity index to identify postoperative recurrence in 60 CD patients. They found that the combination of a 34.5% cut-off of maximum CE with the other US parameters predicted endoscopic recurrence with a 94.4% of accuracy and a good correlation (κ = 0.82; *p* < 0.001). A cut-off >46% CE predicted a moderate–severe endoscopic recurrence (Paredes et al., 2013). Recently, a score combining the US wall thickness, the color Doppler grade and contrast parameters showed an high degree of accuracy in the prediction of endoscopic CD activity. A similar accuracy was maintained even without considering CE results in a simpler version of the score (Ripollés et al., 2021).

However, CEUS has some intrinsic limitations if compared to traditional IUS. The perfusion quantification cannot be compared among different scanners, and both time and costs are higher. Moreover, reproducibility is affected by multiple confounding factors depending on the imaging settings of the available instrumentations, on the operator technique such as the injection method and the pressure exerted by the probe on inflamed bowel wall, and also on patients’ characteristics such as the body habitus, the depth of the region of interest and the gas in gut cavity (Cheng et al., 2016). However, the recent introduction of specific software integrated into US devices such as Qontrast (Bracco, Milan, Italy) and QLAB (Philips, Koninklijke, Belgium) allows a quantitative and semiquantitative analysis of contrast enhancement, limiting the subjective component of the evaluation.

#### Sonoelastography

Sonoelastography is a relatively new sonographic technique that assesses the stiffness of tissues. It is already used in clinical practice for several applications, in particular for breast and thyroid evaluation, and it has been more recently also proposed for assessing gastrointestinal tract. Sonoelastography is able to estimate tissue elasticity by means of US force that propagates a wave into the tissue. Since the wave velocity depends on tissue mechanical properties and mainly on its elasticity, it has been assumed that it is able to provide information on histological features and more precisely on the presence of fibrosis of the bowel wall.

There are two types of elastography: strain and shear wave elastography. With strain elastography compressive force is applied to tissues with repeated pulses to measure lesion stiffness. This can be expressed on a color scale (e.g., from red, soft to blue, hard) for qualitative assessment and/or expressed as wall-to-mesenteric fat strain ratio (i.e., “strain ratio”), for semi-quantitative assessment. In contrast to strain elastography, shear-wave elastography uses an acoustic radiation force impulse, which allows measurement of the propagation speed of shear waves within the tissue. Shear waves propagate faster in hard than in soft tissue and this allows to locally quantify its stiffness. This can be qualitatively assessed by analyzing a colour-scaled image and/or quantitively by determining the maximum elasticity value in either kilopascals (kPa) or meters per second. To date, a limited role is reserved for sonoelastography in the identification of disease activity and inflammation. Only one study assessed inflammation on UC patients, showing a significant correlation between qualitative images of real-time strain-elastography and the severity of lesions at colonoscopy (Ishikawa et al., 2011). However, in more recent studies, Serra et al. and Dillman et al. did not find any significant relationship between elastography parameters and inflammation (Table 1) (Dillman et al., 2014; Serra et al., 2017). Thus, this technique is mainly reserved to the assessment of fibrosis and the differentiation between fibrotic and non-fibrotic stenoses.

**TABLE 1:** Imaging techniques and assessment of inflammation in CD compared with histology (i.e., reference standard: histopathology resection). Adapted from Bettenworth et al. (2019) and Gordon et al. (2020).

StudyYear Type	Pts (n)	Imaging technique	Features	Results


Maconi et al. (2003)	43	US	Evaluation of echo pattern (hypoechoic, stratified or mixed)	Successful differentiation between inflammatory, mixed and fibrotic strictures.
2003				Inflammatory stenoses showed a hypoechoic echo pattern
Prospective				
Ripollés et al. (2013)	25	CDUS	Assessment of:	Successful differentiation in inflammatory and fibrotic strictures
2013		CEUS	-Wall thickness	Transmural complications, color Doppler grade and percentage of increase in contrast enhancement were significantly associated with the pathology inflammatory score (*p* = 0.018, *p* = 0.036 and *p* = 0.005, respectively)
Prospective			-Inflammatory markers: Loss of stratification, transmural complications, lymphadenopathy	Sonographic and pathology scores showed a good correlation in inflammation (Spearman’s, r = 0.53)
			-US Doppler signal	
			-Quantitative CE	
			-Presence of stenosis or prestenotic dilation	
Wilkens et al. (2018)	25	CEUS (+CE MR enterography)	Peak signal intensity, time to peak, area under the time-intensity curve, wash-in rate, wash-out rate, wash-in perfusion index, area under the curve during wash-in and wash-out, fall time, mean transit time	Unsuccessful differentiation in none, mild/moderate to severe fibrosis and inflammation using CE imaging
2018				Successful determination of the degree of inflammation and fibrosis (none, mild/moderate to severe) using US without CE effects
Prospective				Correlation between US wall thickness and histological inflammation (*p* = 0.001)
				No correlation was found between the severity of inflammation or fibrosis on histopathology and on CEUS
Girlich et al. (2011)	20	CEUS	Description of the perfusion pattern of the inflamed bowel wall	Significant correlation with the parameters of the quantitative CEUS analysis
2011			Specific quantification software employ for perfusion assessment	Strong negative correlation (r = – 0.677, *p* < 0.01) between the histopathological score and the time-to-peak (TTP)
Prospective				Strong correlation between TTP and single parameters of the histopathological scoring system (erosion, crypt architecture, crypt, density of eosinophils, excess of intraepithelial neutrophils)
Serra et al. (2017)	26	Strain elastography	Strain ratio measurement	Unsuccessful differentiation in fibrosis and inflammation
2017				No correlation was found between strain ratio and inflammation (*p* = 0.53)
Prospective				
Dillman et al. (2014)	12	Shear wave elastography	Shear wave speed	No significant difference in mean shear wave speed between high–score and low–inflammation score bowel segments
2014				The relationship between bowel wall shear wave speed and inflammation was less correlative and nonsignificant
Prospective				

## Intestinal Ultrasound and Fibrosis

### Applications

Due to the transmural inflammation in CD patients, endoscopic assessment should be always combined with cross sectional imaging diagnostic techniques, which provide a panoramic evaluation of gut, beyond the evaluation of the mucosal surface. ECCO-ESGAR guidelines suggest using an imaging technique, such as IUS, MRE or CT enterography for the assessment of the small bowel activity, for the response to treatment or in case of relapse, persistent disease activity, new unexplained symptoms, before changing in therapies and in postoperative recurrence. The guidelines underlined the role of both IUS and MRE for the detection of small bowel strictures, but also pointed out their inability in the evaluation of the fibrosis. Moreover, they suggest that the choice of the technique should be taken accordingly to the center experience (Maaser et al., 2019).

In fact, according to an observational study involving 71 CD patients, the overall accuracy of US was similar to MRE for the detection of the mural lesions. However, the specificity of US was superior to MRE for bowel wall thickness, loss of wall stratification and stenosis, while the MRE had higher sensitivity in the detection of loss of wall stratification and stenosis. US had higher sensitivity in the detection of ascites, while other mesenteric parameters were similar for both techniques (Yuksel et al., 2019).

#### Identification and Characterization of Strictures

CD is a chronic relapsing inflammatory disease, which evolves in a progressive and irreversible damage of the bowel. According to the Montreal Classification (Sturm et al., 2019), the course of the disease could be penetrating, stricturing or non stricturing/non penetrating. However, this classification is not fixed over time, and the clinical history can change or show mixed types, such as fistula and abscess combined with strictures leading to a high risk of surgery (Gionchetti et al., 2017). This is particularly true for ileal CD, which is usually an inflammatory disease at the time of diagnosis but may become stricturing and penetrating, as well as complicated by strictures and then fistulas and abscesses during the following 10–15 years in almost all patients (Cosnes, 2008).

The stricture, with or without penetrating complication, is still the main indication for surgery, and up to 70% of patients during their life undergo a surgical operation for this complication (Bernell et al., 2000). However, strictures in CD are not always an irreversible condition. The CD strictures show different degrees of inflammation and fibrosis. Rarely, a stricture in CD patients is purely either fibrotic or inflammatory (Rieder et al., 2017).

Since the management and treatment of inflammatory strictures is different from fibrotic strictures, it is useful to differentiate which form is predominant. In particular, inflammation can be successfully treated with medical therapy, such as corticosteroids or targeting TNFα, but fibrosis is an irreversible condition that can be treated only mechanically with balloon dilatation or bowel resection (Gomollón et al., 2017).

Therefore, the differentiation between inflammatory and fibrotic stricture is crucial for the management of CD patients. Unfortunately, no biomarkers, endoscopy or histology could identify the proportion of fibrosis within the stenosis. Likewise, no imaging technique is validated for the identification of the proportion of fibrosis. However, new imaging techniques are emerging and could help in the assessment of the fibrosis within the strictures. In particular, CEUS and Doppler US may evaluate hypervascularity correlated with proportion of inflammation, and sonoelastography, which is a technique that measures the stiffness of tissues, could be used for the identification of fibrosis (Maaser et al., 2019).

The sensitivity and specificity of IUS for the diagnosis of strictures in CD patients range between 80–100% and 63–75%, respectively (Bettenworth et al., 2019), but the differentiation of fibrotic strictures from the inflammatory ones is still a challenge.

The gold standard of the diagnosis of fibrosis remains histology through the analysis of surgical specimens. As reported by Chen et al., smooth muscle hypertrophy and hyperplasia is the most prominent histological change in fibrostenosic bowel strictures. In Crohn’s strictures we can find also neuronal and adipose hyperplasia along with active and chronic inflammation and fibrosis. In particular, in Crohn’s strictures the normal submucosal collagen and adipose tissue are replaced by fibrosis and accompanied by hyperplasia of the muscularis mucosa, the so called muscularization of the submucosa. The muscularis propria is also thickened and expanded by collagen (Chen et al., 2017). All these factors concur to produce the bowel wall thickening detectable by IUS, and in determining the narrowing of the lumen of the bowel. These are inversely correlated, because even only a bit improvement in the thickening suffice to produce a large increase of the luminal area (Yaffe and Korelitz, 1983).

Several studies have been published about the accuracy of imaging techniques to differentiate between fibrotic and inflammatory components of the stricture. However, only few studies compared these techniques with histology (Table 2).

**TABLE 2:** Imaging techniques and characterization of the fibrotic stricture compared with histology.

Study	Year	N pts	Imaging technique	Features	Histopathologic index	Results
Maconi et al. (2003)	2003	43	TUS	Stratified echo pattern or hypoechoic pattern	Fazio et al	Sensitivity 100% specificity 63.3%, PPV 72%, NPV 100% of stratified or mixed echo pattern associated with moderate-severe or intermediate degree of fibrosis in muscolaris mucosae and submucosa
Wilkens et al. (2018)	2018	25	TUS	-Wall thickness	Chiorean et al	-Wall thickness was correlated to fibrosis (r = 0.399, *p* = 0.048)
			CEUS	-Peak enhancement, total area under curve, wash-in and wash-out area under curve, wash-in perfusion index, wash-in rate, rise and fall time, mean transit time local		-No correlation was found between CEUS and fibrosis (r = -0.28, *p* = 0.19)
Ripollés et al. (2013)	2013	25	CDUS	-Stenosis, prestenotic dilatation, color Doppler grade 0 or 1, contrast enhancement <46%	Chiorean et al	-Good correlation between US and histopathological score (Spearman’s, r = 0.50)
			CEUS	-A formula to calculate the percentage of increase in wall brightness and the “time-to-peak”		-Using a cut-off of 65% of enhancement increase, CEUS had sensitivity 93%, specificity 69%, PPV 78%, NPV 90%, accuracy 82%
Baumgart et al. (2015)	2015	10	US-based real-time elastography	RTE strain mean values	Collagen deposits	RTE strain values were significantly higher in unaffected segments compared with affected (*p* < 0.001)
Serra et al. (2017)	2017	26	TUS	-Thickness and stratification score	Chiorean et al.	No correlation was found between thickness score (*p* = 0.198), stratification score (*p* = 0.632), Color-Doppler score (*p* = 0.288), CEUS score (*p* = 0.205) and MSR (*p* = 0.877)
			CDUS	-Color-Doppler score		
			CEUS	-CEUS score		
			RTE	-Mean strain ratio (MSR)		
Chen et al. (2018)	2018	35	SWE	Shear-wave elastography (SWE)	Semiquantitativscoring system	Cut-off of SWE 22.5 kPa had sensitivity 69.6%, specificity 91.7% and AUROC 0.822 to differentiate between mild/moderate and severe fibrosis
Lu et al. (2017)	2017	15	-CEUS	-Peak enhancement	Active and chronic inflammation, fibrosis, and muscular hypertrophy	-Peak enhancement was negatively correlated with fibrosis (r 1⁄4 20.59, *p* 1⁄4 0.02)
			-SWE	-SWE measures		-No significant correlation between SWE and fibrosis (*p* = 0.05)

#### Prognostic Value (Treatment Response)

The assessment of fibrosis on treatment response have been investigated only in one study from Orlando et al. (Orlando et al., 2018). 31 patients without strictures were assessed by strain elastography before a biologic treatment and 3 and 12 months later. Bowel wall thickening and strain ratio have been assessed. The study showed that baseline BWT was not different in patients who responded to the pharmacological treatment and those who underwent operation, while the baseline strain ratio (and so the stiffness of the bowel wall) was significantly greater in patients who underwent operation in the following 2 years and in those who did not reach transmural healing. In particular a SR > 2 was able to discriminate the risk of poor outcome after biologics in the following 2 years (Orlando et al., 2018).

#### Post Operative Recurrence

A large proportion of CD patients, particularly patients with ileal CD, require surgery due to strictures, penetrating disease, or refractoriness to medical treatment. Noteworthy, more than half of them need further surgeries for the same complication (Buisson et al., 2012). To date, the evaluation of the risk of clinical and surgical recurrence for these patients, relies on assessment of presence and severity of endoscopic recurrence by means of Rutgeert’s score, performed within one year after surgery (Gionchetti et al., 2017).

ECCO guidelines have recognized that IUS is a useful emerging tool, alternative to ileocolonoscopy, to identify postoperative recurrence (Maaser et al., 2019). A meta-analysis has shown that the detection by IUS of a pre-anastomic BWT ≥3 mm may detect a postoperative recurrence with an overall sensitivity and specificity of 0.94 (95% CI, 0.86–0.97) and 0.84 (95% CI, 0.62–0.94) and that a post-operative BWT ≥5.5 mm may predict a severe postoperative recurrence (Rutgeert’s ≥ 3) with a sensitivity of 83.8% (95% CI, 73.6–90.6%) and specificity of 97.7% (95% CI, 93–99%) (Rispo et al., 2018).

However, it should be acknowledged this meta-analysis included some studies with a small number of patients and with a very high pretest probability of recurrence, thus making the sensitivity of IUS likely oversized. In addition the studies that assessed the accuracy of IUS before 6 months (e.g. at 3 or 6 months) provided disappointing results.

Overall, IUS is now considered a promising tool for assessing postoperative recurrence and to predict the long term outcome of patients. However, prospective studies using IUS coupled with new technologies (e.g., CEUS or elastography) and combined with fecal calprotectin are awaited to estimate the postoperative risk of stenosis and new surgeries in CD patients. On this regard, it has been demonstrated that in post-operative patients, independently of the time from the previous surgery, a BWT >3 mm may double the risk of surgical recurrence compare to patients with BWT <3 mm, and that the incidence of new surgical interventions is positively correlated with the degree of BWT (Cammarota et al., 2013). Likewise, also in patients who undergo conservative surgery (e.g. stricturoplasties or minimal bowel resections) (Maconi et al., 2001), the behavior of BWT may be a relevant prognostic factor. In fact, the improvement of BWT >2 mm compared to baseline or the normalization of BWT at 6–12 months after surgery has significant favorable prognostic impact on clinical and surgical recurrence, while the IUS detection of unchanged or worsened BWT after surgery predicts a high risk of clinical and surgical recurrence (Maconi et al., 2001). However, the real long term prognostic value of postoperative IUS findings needs further confirmations, as well as the usefulness of therapy escalation, if any, in these patients.

### Techniques and Parameters

IUS can detect strictures as intestinal segments characterized by thickened bowel walls, narrowing of the lumen with proximal bowel dilatation greater than 25 mm (Maconi et al., 1996; Panés et al., 2011; Maconi et al., 2018). In particular, in a recent systematic review, the features used to identify the stricture were at least one among luminal narrowing, wall thickness and prestenotic dilatation. Using only one feature, the sensitivity was 80% and specificity 75%. The SICUS which uses 500–750 ml of PEG oral solution before IUS had sensitivity 88–98% and specificity 88–100% for the diagnosis of the stricture using all three parameters (Bettenworth et al., 2019).

#### B-Mode Ultrasound and Color-Doppler Ultrasound

Regarding the accuracy of IUS in suggesting fibrosis of CD, it has been shown that the thickening of the muscularis propria (clearly visible with IUS), but not the entire bowel wall thickening, was predictive of treatment response and, in particular, of poor response to anti-TNFα therapy (Saevik et al., 2014).

A prospective study including 43 CD patients operated for a single ileal stricture shows that the stratified or mixed echopattern of the wall of the stricture was associated with a moderate-severe or intermediate degree of fibrosis showing a 100% sensitivity, 63.3% specificity, 72% PPV and 100% NPV. The hypoechoic echopattern was more related to the presence of neutrophil infiltrate (Maconi et al., 2003).

Wall thickness measured by IUS was reported associated to fibrosis in a small cohort of CD patients but without a significant correlation between CEUS and fibrosis (Wilkens et al., 2018).

A recent study assessed the utility of mural and extramural sonographic features of CD as markers of inflammation and fibrosis in comparison to histological sections of the bowel of 12 operated CD patients. It shows that mucosal thickness was mainly associated with fibrosis (Bhatnagar et al., 2021).

Another study evaluated the accuracy of US, by using several parameters - such as wall thickness, transmural complications, color Doppler grade, quantitative analysis of the contrast enhancement at CEUS and the presence and severity of strictures - to characterize intestinal inflammation and fibrosis in CD strictures. When compared to the histopathology findings, a negative association was found between the color Doppler grade and the pathologic fibrostenotic score (Ripollés et al., 2013).

#### Contrast-Enhanced Ultrasound

International guidelines recommend the use of CEUS to detect hypervascularity and inflammatory activity of CD strictures (Sidhu et al., 2018). Indeed, the intravenous contrast agent enhances the highly vascularized Crohn’s stenotic segments, which are more likely inflammatory stenosis, and it does not enhance the non-vascularized ones, which are likely fibrotic (Wilson and Burns, 2010).

However, the quantitative assessment of the blood flow within the wall of strictures, inflammatory or fibrotic, may be difficult and not always reliable. Several parameters have been identified to describe and give an estimation of the flow such as the wash in rate, the time to peak, the peak enhancement and the area under the curve (Quaia et al., 2018). Using some of these parameters, the first studies evaluated the blood flow within the wall of the strictures compared to histologic findings, showing positive and very promising results. A small study, including 8 CD patients who underwent CEUS and surgery in the following year, has shown that three patients with fibrotic strictures had no or low signal echo intensity after contrast administration (Kratzer et al., 2002). These preliminary results have been confirmed by another study showing that a 65% of contrast enhancement within the stricture wall had sensitivity, specificity, PPV, NPV and accuracy of 93, 69, 78, 90, and 82% for discriminating predominant inflammatory from predominant fibrotic stenoses (Ripollés et al., 2013). These promising results have been confirmed by Lu et al. who showed an inverse correlation between the peak enhancement at CEUS and the histological degree of fibrosis (Lu et al., 2017), but not by other studies (Serra et al., 2017; Wilkens et al., 2018). In particular, Wilkens et al. used several CEUS parameters to characterize the stricture but did not find any positive or negative correlation between CEUS parameters and the histological features of the strictures. Interestingly, also MRE parameters did not show any positive or negative correlation with histological fibrosis and inflammation (Wilkens et al., 2018).

#### Sonoelastography

An interesting sonographic tool that may be used to characterize the strictures and assess fibrosis is elastography, both strain elastography and shear-wave elastography (SWE). The strain elastography investigates the stiffness of the bowel wall by means of repeated pressures with the probe on the abdominal wall. The system informs the operator when the signal is stable and reliable or reproducible and the image is captured and analyzed. Conventionally, the blue or red color means hard or soft tissue, respectively. The system gives either a qualitative and subjective assessment of the stiffness by examining the colors, and a quantitative estimation of the stiffness, by comparing the value within a region of interest in the wall with a similar one outside of the wall, usually taken from the softer tissue around (Stidham et al., 2011; Lu et al., 2019; Vestito et al., 2019). On the contrary, the SWE is a system that directly calculates the elasticity in kPascal thanks to the diffusion of the beam within the tissue in specific regions of interest, without the necessity to pressurize repeatedly the abdomen (Vestito et al., 2019).

A meta-analysis, whose conclusion has some limitations due to high heterogeneity and patient selection between the six studies included, shows that sonoelastography could be useful to detect fibrosis in CD strictures. Three studies used strain elastography and showed that the strain ratio was higher in strictures with fibrosis compared to strictures without fibrosis (*p* = 0.05). Similar results were found by the other three studies that used SWE, where the strain value was statistically higher in fibrotic stenosis (*p* = 0.08) (Vestito et al., 2019).

A few studies have also compared the sonoelastography results with histological evaluation. Fraquelli et al. assessed the ileal strictures in Crohn’s patients by strain elastography, comparing the results with the rate of fibrosis assessed by histology. The ileal wall fibrosis percentages in histological sections showed a significant and positive correlation with ileal wall strain ratios obtained by elastography (*p* = 0.005) (Fraquelli et al., 2015).

A small study, including 16 CD patients with either ileocolonic localization or postoperative recurrence, showed that fibrotic strictures had a significantly higher axial-strain sonoelastography scores than inflammatory strictures (Sconfienza et al., 2016). Chen et al. performed SWE in 35 CD patients who underwent surgery within one week and demonstrated that SWE was significantly higher in patients with fibrosis in the stricture than in patients with inflammation. Setting 22.55 kPa as the cut-off value, the sensitivity was 69.6%, the specificity reached 91.7% with the area under the ROC curve (AUROC) of 0.822, thus allowing to differentiate between mild/moderate and severe fibrosis (Chen et al., 2018).

However, not all the studies showed positive and promising results. Lu et al. did not find a significant correlation between SWE and fibrosis (*p* = 0.05) (Lu et al., 2017), but reported a moderate correlation between SWE and muscular hypertrophy (r 1⁄4 0.59, *p* 1⁄4 0.02) in 15 CD patients. Serra et al. reported data from 26 CD patients who underwent IUS, color-Doppler, CEUS and real time elastography, without finding any correlation between any of the previous techniques and fibrosis score at the histological examination (Serra et al., 2017).

## Conclusion

Despite the high accuracy of US in detecting strictures, and the promising results obtained with the novel sonographic techniques such as CEUS and sonoelastography, several limitations are still present and more studies are needed before using these technique in routine clinical practice and trials.

Ultrasound is a well-known operator and patient dependent technique and, apart from operator experience, there are bowel segments that are difficult to scan by IUS, such as proximal ileum, jejunum and deep pelvic loops due to intestinal gaseous content, which is rather frequent in patients with chronic strictures. In addition, the fine evaluation of long strictures and multiple strictures may be difficult, particularly in case of quantitative assessment with CEUS and elastography because of need of repeated measurements, and the uncertainty on the exact part of the stenotic segment that should be evaluated. On this regard, MRE, which offers a multiparametric more panoramic view, may be more accurate.

Like MRE, the bowel evaluation with IUS currently allows a multiparametric approach where B-mode, Doppler, CEUS and elastography can be applied to simultaneously assess inflammation and fibrosis in the same segment. A preliminary experience on this multiparametric approach has been performed using in combination IUS, CEUS, and sonoelastography by two readers providing an accuracy of 70 and 75% with the area under the ROC curve (AUROC) of 0.953 and 0.921 and both were significantly higher than the single technique in the detection of fibrosis (*p* = 0.001 and *p* < 0.05, respectively) (Quaia et al., 2018).

In the last years, important steps have been done in this direction. First, a valid gold standard for fibrosis and inflammation, which is mandatory to have reliable studies, has been defined. Important systematic reviews and research groups have appropriately addressed this point (Bettenworth et al., 2019; De Voogd et al., 2020). Then, the concept has emerged that the IUS parameters and scores obtained by each technique need a validation and should be reproducible. This has been done in few studies including this review, but the big challenge remains to assess CD activity (Novak et al., 2020), an issue that will be faced for strictures in the near future.

If these criteria will be fulfilled by IUS and MRI, we will have powerful tools to study strictures and fibrosis of CD, we will able to select the most appropriate diagnostic techniques for our patients, reliable to decide if a stricture should be surgically or medically treated and to monitor its outcome after the therapy.
